# Oral Infections and Systemic Health – More than Just Links to Cardiovascular Diseases

**DOI:** 10.3290/j.ohpd.b1993965

**Published:** 2021-09-11

**Authors:** Jukka H. Meurman, Antonio Bascones-Martinez

**Affiliations:** a Professor and Head Physician, Department of Oral and Maxillofacial Diseases, University of Helsinki and Helsinki University Hospital, Finland. Conceptualisation, literature analyses and evaluation, wrote the manuscript.; b Professor, Complutense University, Madrid, Spain. Conceptualisation, literature analyses and evaluation, wrote the manuscript.

**Keywords:** oral infection, periodontitis, systemic health

## Abstract

**Purpose:**

During the past 20 years, a plethora of research reports has been published showing a statistical association between poor oral health and cardiovascular diseases. The aim of this narrative review was to focus on associations between oral infections and non-atherosclerosis-related systemic diseases.

**Materials and Methods:**

An open literature search and evaluation of articles were conducted on Medline and Cochrane databases with the key words ‘oral infection’, ‘periodontitis’, ‘pneumonia’, ‘osteoarthritis’, ‘rheumatic diseases’, ‘inflammatory bowel disease’, ‘kidney disease’, ‘liver diseases’, ‘metabolic syndrome’, ‘diabetes’, ‘cancer’, ‘Alzheimer’s disease’. Cardiovascular diseases were excluded from the analysis.

**Results:**

The scarcity of controlled studies did not allow conducting a systematic review with meta-analysis on the topics, but dental infections have been shown be associated with several general diseases also beyond the atherosclerosis paradigm. However, there is no causal evidence of the role of dental infections in this regard. Poor oral health has nevertheless often been observed to be associated with worsening of the diseases and may also affect treatments.

**Conclusions:**

Maintaining good oral health is imperative regarding many diseases, and its importance in the daily life of any patient group cannot be over emphasised.

Wherever in the body, infections cause upregulation of chemokines, cytokines and other inflammatory mediators, resulting in inflammation. Dental and oral mucosal infections are highly prevalent in populations, and often chronic microbial infections trigger inflammation first in the adjacent tissues. The spread of microorganisms from the mouth causes subsequent local infections in the jaws, facial structures, parapharyngeal and peritonsillar spaces, but may spread even further, e.g. intracranially, or by aspiration to the lungs. All these can have life-threatening consequences.

In addition to direct spread and focal metastatic extra-oral infections, other detrimental systemic effects of oral infections include reactions caused by bacterial metabolites (toxins) and immunological factors (e.g. *Streptococcus viridans* endocarditis and glomerulonephritis); blood coagulation may also be affected. The low-grade general inflammation triggered by oral infections may indeed explain many organ-level problems and complications.^35,66^ From a more general perspective, it has been estimated that at least 20% of small molecules in blood are of microbial origin.^70^ The oral cavity harbours thousands of microbial species and in a dentate person with gingivitis the area of wound is approximately the size of a human hand. Thus, through inflamed periodontal tissues, oral microorganisms obtain direct access to the lymphatic and circulatory systems.^26^ Consequent reactions include metabolic activation of epithelial cells, B- and T-lymphocytes; DNA and its repair mechanisms are also affected.^49^

Most studies linking oral infections to systemic health show statistical associations; mainly cardiovascular diseases (CVD) have been investigated in this context. However, our knowledge of the underlying mechanisms is still poor, despite extensive study. Possible causality remains to be elucidated.

Associations between oral infections and CVDs were not included in this review, because they have been thoroughly discussed elsewhere.^2,38^ Today, data are available which show a distinctly elevated risk in this regard.^1,35^ However, less interest has been given to the potential correlation of oral infections with systemic diseases. Hence, the aim of this study was to explore the pertinent literature in this context. We anticipated finding associations such as those found with CVDs with many other general diseases, in which poor oral health is an additional risk. Examples of the non-CVDs discussed here, which may be associated with oral infections, are given in Table 1.

**Table 1:** Non-cardiovascular systemic diseases for which a link to oral infections has been suggested*

Disease	Reference
Pneumonia	Scannapieco, 2006
Osteoarthrosis	Temoin et al, 2015
Rheumatic disease	Helenius et al, 2005
Crohn’s disease	Halme et al, 1993
Kidney disease	Fisher et al, 2010
Liver disease	Helenius-Hietala et al, 2013
Metabolic syndrome	Hyvärinen et al, 2015
Cancer	Söder et al, 2015
Alzheimer disease	Dioguardi et al, 2020

*One reference example of each entity is given.

## Materials and Methods

This open literature review was based on the key words ‘oral infection’ ‘periodontitis’, ‘systemic health’, ‘pneumonia’, ‘osteoarthritis’, ‘rheumatic diseases’, ‘inflammatory bowel disease’, ‘kidney disease’, ‘liver diseases’, ‘metabolic syndrome’, ‘diabetes’, ‘cancer’, ‘Alzheimer’s disease’. The search in PubMed and Cochrane databases resulted in hits ranging from 187 (Alzheimer’s disease) to 18,759 (cancer), but when only controlled studies were selected, 304 hits remained. The search was conducted in January 2021. The articles were scrutinised jointly by the authors with focus on the controlled investigations. However, no systematic review could be conducted due to the paucity of properly controlled studies.

## Results

The main findings from the searched literature on the associations between oral infections and non-CVDs are given in Table 1. In the following, we present the findings by disease.

### Pneumonia

It has been estimated that close to 500 million cases of pneumonia were encountered in 2019.^19^ Pneumonia is a well-known complication and threat particularly among the frail and it is often the imminent cause of death. Nosocomial pneumonia shows a mortality range from 30% to 70%.^58^ It is easy to understand how oral microorganisms can be translocated to the bronchial tree by accidental aspiration, which subsequently elevates the risk of pneumonia. The risk becomes marked among patients with impaired swallowing and/or cough reflex.^74^

A systematic review by van der Maarel-Wierink et al^80^ concluded that good oral hygiene is essential in preventing pneumonia: ‘According to the results of the current systematic literature review oral health care, consisting of tooth brushing after each meal, cleaning dentures once a day, and professional oral health care once a week, seems the best intervention to reduce the incidence of aspiration pneumonia’.

Community-acquired pneumonia is indeed prevalent among the frail and elderly in particular. Aspiration pneumonia in connection with medical care, such as ventilation, is a significant problem.^17^ On the other hand, studies have clearly shown that treatment of periodontitis reduces the risk of respiratory complications.^75^ As an example, the case-control study by Gomes-Filho et al^23^ showed that periodontitis patients were three times more liable to develop pneumonia than those without periodontitis (odds ratio [OR] 2.88; 95% confidence interval [CI] 1.59 to 5.19). Hence, there is no doubt that maintenance of good oral hygiene, and diagnosing and treating periodontitis in particular, is a necessity in patients with risk of pneumonia or other lower respiratory tract diseases. Nevertheless, a Cochrane review a few years ago concluded that more studies are needed in this area before the evidence can be considered conclusive.^45^

### Osteoarthritis

Musculoskeletal disorders in general were responsible for 150 million disability-adjusted life years (DALY) in 2019, where osteoarthritis alone plays a role with 18.9 million DALYs.^19^ Although the role of oral infections in the aetiology of osteoarthrosis is not known, dental problems have long been associated with endoprosthesis infections.^44^ Dental foci as the source of these infections are rare, but periodontal bacteria have been detected in joint aspirates, suggesting potential haematogenic spread from the mouth.^15,78^ Antibiotic prophylaxis in endoprosthesis surgery is the practice in many countries in cases where dental problems have not been treated, such as in emergency operations. This practice, however, is not evidence based and has also been questioned.^42^

Microorganisms from the oral cavity may nevertheless play a role in the development of arthritis.^47^ Recently, a large study from Korea^39^ showed a statistically significant association between periodontitis and radiographic signs of knee osteoarthritis, with OR 1.25 (CI 1.05–1.49); when the severity of periodontitis increased in the study cohort, the probability of having osteoarthrits also increased. However, more data are necessary to draw a final conclusion.

### Rheumatic Diseases

Rheumatic diseases are manifold, affecting collagen metabolism and consequent symptoms and signs in many organs. In 2019, the systemic autoimmune disorder rheumatoid arthritis (RA) was responsible for 3.26 million global DALYs, accounting for 0.1% of the total burden.^19^ From the oral health point of view, Sjögren’s syndrome has been most extensively studied because of the characteristic hyposalivation, which causes marked oral health problems.^16^ However, Sjögren’s syndrome will not be further discussed in this article because of many specific studies on this topic.^9^

Triggering factors for RA have been intensively researched, and periodontitis is suggested to be among them.^28,37^ There seem to be common risk factors or common pathogenic pathways in these two diseases, shared genetic factors and external factors, such as obesity, smoking and socioeconomic status.^40^ RA and periodontitis also have similarities in morphology and histopathology; oral bacterial genes have been identified from serum and synovial fluid of the patients.^52^ The periodontal bacterium *Porphyromonas gingivalis* has been associated with exacerbation of rheumatoid arthritis.^48^ This bacterium is capable of citrullination of proteins, which generates antibodies associated with the pathogenesis of RA.^41^ Hence, there may indeed be common inflammatory pathways between these two diseases.^6^

Since the presence of high numbers of periodontal pathogenic bacteria may play a significant role in the citrullination process, preventive and therapeutic measures to control both inflammation and microorganisms seem reasonable in the management of RA.^22,59^ In fact, these measures should probably be implemented before RA treatment, as indicated by a retrospective study of 54 RA patients receiving biological disease-modifying anti-rheumatic drugs (DMARD).^53^ In this study, RA patients showed a significant correlation between baseline periodontal inflammation, evaluated by the periodontal inflamed surface area, and the clinical activity of RA, swollen joint counts, and patient’s and evaluator’s global assessment. Moreover, in an interventional study, a substantial number of patients with severe periodontitis and RA did not respond to several rounds of synthetic and biological DMARDs.^55^ However, in 5 out of 8 of those non-responding patients, there was a good response in the clinical DAS-28 scoring of disease activity following non-surgical periodontal therapy. This indicates that a healthy periodontal status may be important for the comprehensive treatment of RA.

An interesting question is how modern biological drug treatment of rheumatic diseases affects the mouth and vice versa. Äyräväinen et al^3^ followed a group of RA patients undergoing different drug treatments but found no association between the anti-rheumatic treatment and periodontal parameters. In that 16-month study, biological, synthetic and conventional disease-modifying antirheumatic drugs were used in patients with early vs chronic disease. However, in the same study, RA patients already in the early phase of the disease had poorer oral health compared with controls, and a positive association was found at the follow-up between dental disease indices and the activity of RA (p < 0.001).^4^ These questions also call for future elaboration. Preferably case-control studies with enough power and long observation times should be conducted in order to verify the possible causal role of oral infections in rheumatic diseases.

### Inflammatory Bowel Diseases

The epidemiology of inflammatory bowel diseases (IBD), Crohn’s disease and ulcerative colitis shows that in Europe and North America the incidence of Crohn disease varies between 3 and 15/100,000 persons per year and that of ulcerative colitis from 2 to 15/100,000. It is noteworthy that the incidence figures have increased in the 21st century.^54^ The aetiology of IBD is not known, but there is a strong autoimmune component. Dental infections have been observed to be associated with the exacerbation of Crohn’s disease; thus also a local infection/inflammation with systemic spread of microorganisms may play a role in the aetiology and progression of these diseases.^25,51^ Periodontitis in particular seems to be linked to IBDs.^81^

IBDs have indeed been shown to express local hypersensitivity towards endogenic infection. In his review article, Brandtzaeg^5^ states that perturbation of a tightly controlled cytokine network, with abnormal crosstalk between several cell types, may explain the immunopathology of chronic inflammatory mucosal diseases, whether IBD or periodontitis. Thus, there may be a common denominator in the susceptibility to oral infections and IBD.

Among the IBDs, it should be borne in mind that Crohn’s disease also has oral manifestations, such as granulomatous lesions in oral mucosa.^65^ Discussing these, however, is beyond the scope of the present article. Finally, treatment of IBD may also affect the oral cavity and have consequences for dental care.^57^ One example is immunosuppressive medication often used by IBD patients.

### Kidney Diseases

Kidney diseases are prevalent in populations world wide, and chronic kidney disease (CKD) was responsible for 41.5 million DALYs in 2019.^19^ Dental diseases are also prevalent among patients with CKD. For example, a systematic review reported that periodontal disease was more common in patients with severe kidney disease compared with those with less severe disease (56.8% vs 31.6%).^72^ Furthermore, the treatment of periodontitis has been shown to positively affect the glomerular filtration rate of affected patients.^8^ Patients with diabetic nephropathy have been shown to be particularly susceptible to dental infection-derived problems.^61^ This is important to keep in mind, since diabetic nephropathy is the leading cause of CKD in many countries.^56^

**Fig 1:**
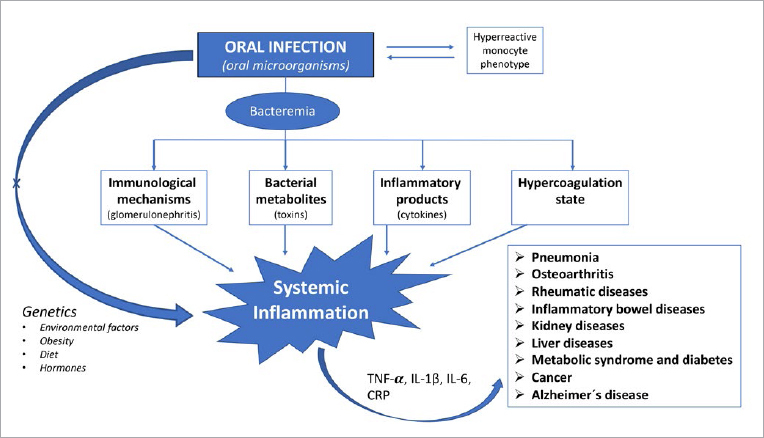
Upregulated inflammatory mediators, cytokines and other pathological reactions are the principal mechanisms linking oral infections to a number of systemic diseases.

Ruokonen et al^71^ reported on 144 chronic kidney disease patients followed from the pre-dialysis stage up to kidney transplantation, and observed a significant difference in mortality of patients who had had diabetic nephropathy compared with those with other causes of CKD. Survival was 23.8% in the diabetic nephropathy group vs 59.9% in those whose disease had other causes (p<0.001).^71^ The interaction between oral infections and many systemic diseases, such as kidney diseases, is based on the ability of oral microorganisms to translocate and upregulate a number of inflammatory mediators (Fig 1) both locally and in the blood. These mechanisms in turn may be directly responsible for the effects observed.^30^

### Liver Diseases

Cirrhosis and other chronic liver diseases account for 46.2 million global DALYs in 2019, and 12.5. million DALYs were due to liver cancer alone.^19^ From the oral health perspective, a special and important treatment group consists of those with a liver transplant (LT). Before a patient can be admitted to the waiting list for LT, oral infection foci must be diagnosed and treated because of the risk of post-transplant infectious complications. A significant association has been shown between post-LT systemic infections and lack of pre-transplant dental treatment.^27^ On the other hand, chronic liver diseases can have adverse effects on oral health.^21^ Caries and periodontitis are the most common oral diseases among patients with chronic liver diseases.^29^

Hyposalivation is another problem among LT candidates, promoting the accumulation of dental plaque and increasing the risk for oral infections.^24^ In particular, LT recipients with an underlying indication of chronic liver disease had more dry mouth-related symptoms compared to healthy controls and patients with acute liver failure.^29^ Hyposalivation diminishes the individual’s quality of life, causing difficulties in eating, tasting, speaking, and wearing prostheses.

Systemic spread of dental infections seems to correlate with accelerated liver disease.^79,84^ A recent study has shown that periodontitis was an independent risk factor for severe chronic liver disease.^29^ Furthermore, the severity of liver disease has been associated with poor oral health in general.^1^ In particular, LT recipients have been reported to have poor oral hygiene and a high need for dental and periodontal treatment.^36^

### Metabolic Syndrome and Diabetes

Metabolic syndrome and type 2 diabetes have been called the new epidemic because of ever increasing obesity in the world population. Diabetes alone was responsible for 2.6% of global DALYs in 2019.^19^ Again, like with many systemic diseases, periodontal disease is also associated with metabolic syndrome.^43^ A meta-analysis by Nibali et al^60^ showed an OR 1.71 (CI 1.42–2.03) for the association between periodontitis and metabolic syndrome in a total of 36,337 subjects. More recently, a systematic review and meta-analysis by Gobin et al^20^ of 39 articles calculated an OR 1.99 (CI 1.75–2.25) for the association between periodontitis and metabolic syndrome. Minor differences were observed between studies from different populations, but in all cases the results were statistically significant.^20^

The possible link may be a common inflammatory pathway and advanced glycation end products.^67^ Both diseases result from the confluence of various triggering and modifying factors, and there are interindividual differences in the risk of their development. Oral infections in general affect endothelial function with consequences for sugar metabolism as well.^34^ Metabolic syndrome is a globally increasing, multifaceted problem due to obesity, insulin resistance, and genetic background factors, leading to type 2 diabetes and cardiovascular disease.^73^ Diabetes and oral infections seem to be a two-way street, where infections on the one hand and diabetic susceptibility on the other intertwine. There are numerous studies assessing the role of oral infections in diabetes and, vice versa, the effect of diabetes on oral health parameters.^18^

Diabetes mellitus is, as previously stated, a systemic disease associated with serious complications that can affect the quality of life and life expectancy of the patient.^32,68^ On the other hand, control of periodontal disease may enhance glycemic control in patients with type 2 diabetes. In turn, improved glycemic control may contribute to better control of periodontal disease.^10^ Given the interrelationship between diabetes and periodontal disease, it is important to establish good communication between the specialist responsible for a diabetic patient and the patient’s dentist. Further details of these interactions are available in special articles on this topic.^11,31^

### Cancer

Cancer is the second leading cause of death worldwide, responsible for 9.6 million deaths in 2018.^83^ Tobacco use is the major risk factor, but infections are also estimated to play a role in up to 25% of all cancers, in particular in the developing countries. Infectious agents commonly mentioned in this context are hepatitis and Epstein-Barr viruses, human papilloma virus (HPV), and the bacterium *Helicobacter pylori*.

The association between certain types of HPV with oropharyngeal cancer is well established and shall not be discussed further in the present article.^7^ Evidence of the association between oral bacteria/yeasts and cancer is not as strong as is the case with specific viruses; nevertheless, there seems to be a link particularly between periodontal disease and cancer in general.^49^ Söder et al^77^ presented data from Sweden linking gingival inflammation to any cancer. The same group of researchers also observed that poor oral hygiene as measured by high dental plaque index was statistically significantly associated with cancer mortality.^76^ Further, from the same cohort, the research also reported how dental infections were associated with cancer in periodontally healthy subjects.^82^ In that study, the proxy for dental infections was the number of missing molars where, in particular, a missing second mandibular molar was associated with cancer (OR 2.62; CI 1.18–5.78). This result can be explained by the common professional wisdom which holds that teeth are mostly extracted due to severe caries or periodontitis, so that missing teeth may indeed indicate past dental infections. Overall, the role of oral microbiota in the development of malignancies is an interesting question, but so far the evidence remains weak.

### Alzheimer’s Disease

Alzheimer’s disease is a neurodegenerative disease responsible for most dementia. Age and heredity are the principal risk factors, resulting in altered metabolism of amyloid precursor protein and subsequent deposition of ß-amyloid plaques in the brain. It has been suggested that inflammation in the central nervous system could trigger this development.^33^ Alzheimer’s disease is responsible for about 1% of global DALYs (25.3 million) according to the latest statistics.^19^

A number of studies have been published which aimed to investigate the association between periodontitis and Alzheimer’s disease. Periodontitis, being a highly prevalent chronic inflammatory disease, might – by upregulating cytokines and inflammatory mediators (Fig 1) – also increase the cerebral inflammatory stage, thus affecting pathogenic pathways leading to Alzheimer’s. Periodontal pathogens, particularly *P. gingivalis*, have also been linked to the development of dementia; traces of this bacterium have been found in brain tissue of patients who died with Alzheimer’s disease.^69^ *P. gingivalis* and its metabolites gingipains have been detected in brain tissue of Alzheimer patients.^14^ It also possible that the bacterium contributes to intracerebral amyloid-β accumulation, which is one of the putative mechanisms in the development of dementia.^63^ Numerous microorganisms have indeed been suspected to play a role in the development of Alzheimer’s disease via the inflammatory ß-amyloid pathway.^62,64^ However, the pathologic process is slow, and it has been estimated to take up to 20 years before manifest symptoms appear.

Recently, Dioguardi et al^13^ published a systematic review on this topic, concluding that there is nevertheless not enough evidence to consider periodontitis as a risk factor for Alzheimer’s disease. In the voluminous literature they searched, only 15 articles fulfilled the criteria for closer analysis. Based on these, the authors stated that in future studies, the effect of reduction of local inflammation, such as periodontitis, and establishing the possible role of periodontal bacteria in the pathogenesis of Alzheimer’s disease should be investigated.^13^

## Discussion

Investigating the associations of oral infections with the nine disease entities discussed here clearly showed that poor oral health is important not only as a possible risk factor in the development and progression of a disease but also when considering treatment. In practice, when thinking about the aetiology and pathogenesis of any systemic disease, the eventual role of hidden infections that often have no subjective symptoms, as is the case with many oral infections, must not be forgotten. Dental infections must therefore be diagnosed and appropriately eradicated before commencing treatment of the underlying systemic disease. Immunosuppressive therapy especially, or any major surgery, are good examples of this. The common denominator of the associations discussed here is the chronic and mostly subclinical systemic infection that is triggered by an oral/dental infection and thus indirectly affects all parts of the body through blood circulation.

Finally, a few words need to be said about the strengths and limitations of the current literature review. This was to our understanding the first comprehensive review on the associations between oral infections and the nine non-CVDs. Here, only weak evidence was found. Secondly, lack of controlled and long-term follow-up studies did not allow any conclusions about causality, and it was not possible to follow the systematic review principle and conduct meta-analyses with this material. Consequently, the research community should commence properly controlled series with enough statistical power and sufficiently long follow-up times with the different patient materials. These studies should be carried out as multi-centric investigations, an approach that is commonly lacking in the dental literature. However, it is clear that oral infections are linked to many systemic diseases beyond the atherosclerosis spectrum.

## Conclusion

Since the late 1980s, dental research has been particulary focused on systemically investigating the association between periodontitis and CVD. This statistical association was approved by the American Heart Association.^46^ However, many other systemic diseases are also associated with poor oral health, as briefly reviewed in this article. Unfortunately, the studies on the associations between oral infections and non-CVDs mostly investigated a low number of patients, and hardly any long follow-up studies have been published. Therefore, it was not possible to follow the systematic review principle and conduct meta-analyses with this material. However, maintaining good oral health is imperative regarding many systemic diseases and its importance in daily life of any patient group cannot be over-emphasised. Prevention of dental infections by maintaining good daily oral hygiene is, of course, the best approach, which may also ameliorate the burden of many systemic diseases. Furthermore, frequent consultations between the patient’s physician and dentist should be encouraged; this is particularly important when treating medically compromised patients.
